# Characterizing Patient Phenotypes and Emerging Trends in Application of Spatial Analysis in Individual-Level Health Data

**DOI:** 10.21203/rs.3.rs-3443865/v1

**Published:** 2023-10-16

**Authors:** Abolfazl Mollalo, Bashir Hamidi, Leslie Lenert, Alexander V. Alekseyenko

**Affiliations:** Medical University of South Carolina; Medical University of South Carolina; Medical University of South Carolina; Medical University of South Carolina

**Keywords:** clinical phenotypes, electronic health records, geocoding, geographic information systems, patient phenotypes, spatial analysis

## Abstract

**Objectives:**

Health information systems (HIS) commonly contain patient addresses that provide valuable data for geocoding and spatial analysis, enabling more comprehensive descriptions of individual patients in biomedical studies. Despite the widespread adoption of HIS, no systematic review has examined the extent to which spatial analysis is used in characterizing patient phenotypes.

**Materials and Methods:**

We systematically evaluated English-language peer-reviewed articles from PubMed/MEDLINE, Scopus, Web of Science, and Google Scholar databases from inception to August 20, 2023, without imposing constraints on time, geography, or specific health domains.

**Results:**

Only 62 articles met the eligibility criteria. These articles utilized diverse spatial methods, with a predominant focus on clustering techniques, while spatiotemporal analysis (frequentist and Bayesian) and modeling were relatively underexplored. Geographically, the use was limited, involving only nine countries, with over 80% of studies conducted in the United States. Moreover, a noteworthy surge (82.3%) in publications was observed post-2017. The publications investigated various clinical areas, including infectious disease, endocrinology, and cardiology, using phenotypes defined over a range of data domains, such as demographics, diagnoses, and visit. The primary health outcomes investigated were asthma, hypertension, and diabetes. Notably, patient phenotypes involving genomics, imaging, and notes were rarely utilized.

**Discussion and Conclusion:**

This review underscores the growing interest in spatial analysis of HIS-derived data and highlights knowledge gaps in clinical health, phenotype domains, geospatial distribution, and spatial methodologies. Additionally, this review proposes guidelines for harnessing the potential of spatial analysis to enhance the context of individual patients for future biomedical research.

## INTRODUCTION

Health information systems (HIS) have significantly enriched clinical research by providing relatively cost-effective, time-efficient, and convenient sources of a large population of patient records [1, 2]. Because HIS often contain patient addresses, geographic information systems (GIS) can enable value added analyses via high-resolution geocoding. The simplest of such analyses may be mapping, which for example can promote better understanding of health disparities. Further, patient geocoding can provide a means for linkage of external data such as environmental, demographic, and socio-economic factors for more refined patient phenotyping and a more profound understanding of patient exposures [3].

The possibilities for applying spatial analysis of individual-level HIS-derived data are beyond geocoding, basic mapping, or external data linkage. For instance, spatial network analysis examines proximity to the sources of pollution [4], measures accessibility to healthcare facilities [5], and optimizes resource allocations to mitigate health disparities [6]. Spatial clustering pinpoints statistically significant spatial and spatiotemporal hotspots and cold spots [7], especially when considering longitudinal data. Moreover, spatial and spatiotemporal modeling can identify localized patterns, trends, and relationships within a specific region [8].

While spatial methodologies have the potential to better describe the context of individual patients in biomedical studies, there is a need for improvement in their utilization to derive meaningful insights. To accurately address medical conditions, identify a disease in a patient, and scale that to cohorts of patients, phenotyping is required [9]. Phenotypes are a combination of observable traits, symptoms, and characteristics. They can contain inclusion and exclusion criteria (e.g., diagnoses, procedures, laboratory reports, and medications) and can be used to recruit patients who fit the necessary criteria for clinical trials.

This is the first comprehensive study that systematically reviews the literature using spatial analysis for analyzing HIS-derived data, including electronic health records (EHR), electronic medical records (EMR), electronic patient records (EPR), enterprise data warehouses (EDW), and research data warehouses (RWD), in characterizing patient phenotypes. This review collates and synthesizes existing literature that employed individual-level health data from the above-mentioned HIS in conjunction with advanced spatial analyses and patient phenotyping. Thus, the main objectives of this review are:

1. To evaluate the degree to which advanced spatial methods are currently being utilized with individual-level data sourced from HIS;
2. To identify areas of spatial analyses most applicable to biomedical studies;
3. To categorize publications concerning their biomedical and clinical areas and the specific patient phenotypes they target.

## METHODS

This systematic review was performed using the protocols outlined by the Preferred Reporting Items for Systematic Reviews and Meta-Analyses (PRISMA) to identify the articles that satisfy the eligibility criteria for subsequent data extraction and synthesis.

### Data source

A comprehensive search for peer-reviewed articles was carried out using abstracts and titles screening within PubMed/MEDLINE, Scopus, and Web of Science databases using the search terms in Supplementary Appendix S1. The search was conducted on August 20, 2023, without limitations on time, geography, or specific health domains.

### Search strategy

The initial search comprised two main categories. The first category included a broad set of key terms related to spatial analysis. The second category employed the key terms associated with health information systems. The Boolean operator (AND) was applied to synthesize the two categories.

### Study selection

The retrieved abstracts and titles were imported into Covidence systematic review software (https://www.covidence.org/), where duplicate records between original databases are automatically eliminated. Two reviewers (AM and BH) independently assessed the eligibility of the articles based on the following inclusion and exclusion criteria:

The articles were eligible for primary inclusion if they (1) were composed in English, (2) were original peer-reviewed articles, (3) used individual-level patient data, (4) incorporated at least one form of the spatial method, (5) utilized EHR/ EMR/ EPR/ EDW/ RDW derived data. Conversely, the articles were excluded if they (1) were not peer-reviewed (e.g., letters, editorials, reviews, case reports, abstracts, and grey literature), (2) did not utilize individual level EHR/ EMR/ EPR/ EDW/ RDW data, (3) solely geocoded addresses or generated basic visualizations (e.g., dot map and choropleth map) without any spatial analysis.

The reviewers (AM and BH) independently reviewed the full texts of all remaining articles. The articles were excluded if they lacked phenotype characteristics of patients. Further, we manually checked the references for all the selected articles for possible inclusion. We also searched the first 20 pages of Google Scholar for potential inclusions. A third reviewer (AVA) was consulted to break ties.

### Data extraction

Upon identifying articles that satisfied all inclusion criteria, two reviewers (AM and BH) extracted the following items for each article: title, publication year, country and region, sample size, study period, spatial methodologies, and key findings from the spatial methods. Moreover, articles were assessed to identify clinical domains (including primary and secondary when applicable), health conditions or problems, and themes, including social determinants of health (SDOH), environmental factors, ecological aspects, climate, microbiome, genomics, and clinical phenotypic characteristics. Previous publications have emphasized the importance of data domain sources in phenotyping, underscoring the need for validating the created phenotype [10] and using multiple data sources. Thus, in cases where the included publications did not provide details of data sources but instead referenced previously published works, referenced publications were reviewed. We also documented the number of organizations contributing data. When data originated from multiple hospitals operating within a single hospital system, we treated it as a single organization. Additionally, we cataloged the types of HIS that served as the sources.

### Narrative synthesis

Following data extraction, the articles were categorized into the following spatial methodology classifications: descriptive, clustering, modeling (frequentist), spatiotemporal (frequentist), and Bayesian. The phenotype characteristics were extracted and recorded as free text. It should be noted that the categories were not mutually exclusive.

The quality appraisal of the studies was not feasible due to the substantial heterogeneity in spatial methodologies and health domains. The geospatial distribution of the included studies was visualized using ArcGIS Pro software 3.0 (ESRI, Redlands, CA, US).

## RESULTS

### Study selection

**Figure 1:**
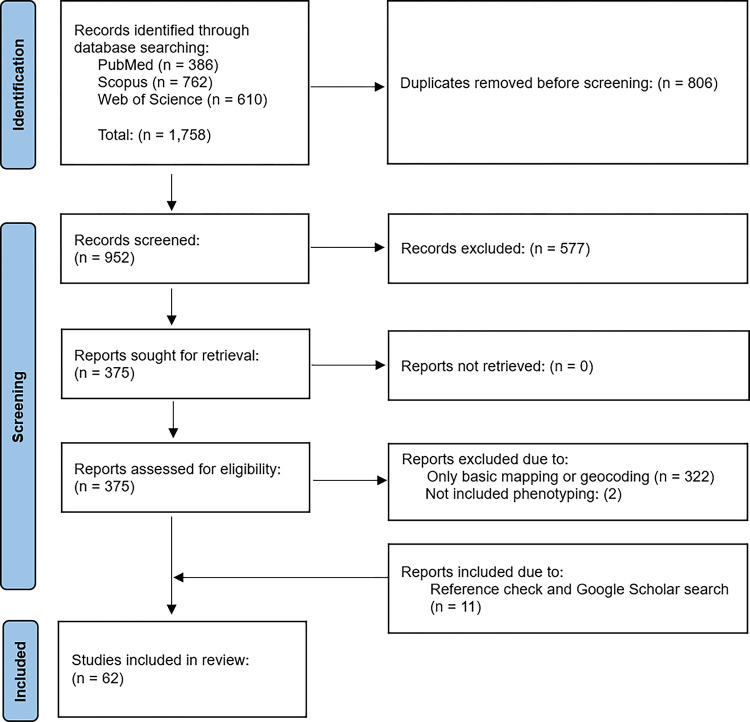
PRISMA study selection flowchart

The initial search yielded 1,758 references. After removing duplicate records, we identified 952 articles for abstract and title screening, from which 375 were selected for full-text review. Out of these, 322 articles were excluded as they only contained geocoding or basic mapping without any spatial analysis. Additionally, two articles were omitted due to the absence of patient phenotype characteristics. We further manually searched references and Google Scholar and found 11 new articles that met the eligibility criteria. Therefore, 62 articles that fulfilled the inclusion criteria were retained for data extraction and synthesis. Figure 1 depicts the PRISMA flowchart for the study selection process.

#### Temporal and geographic distribution of studies

**Figure 2:**
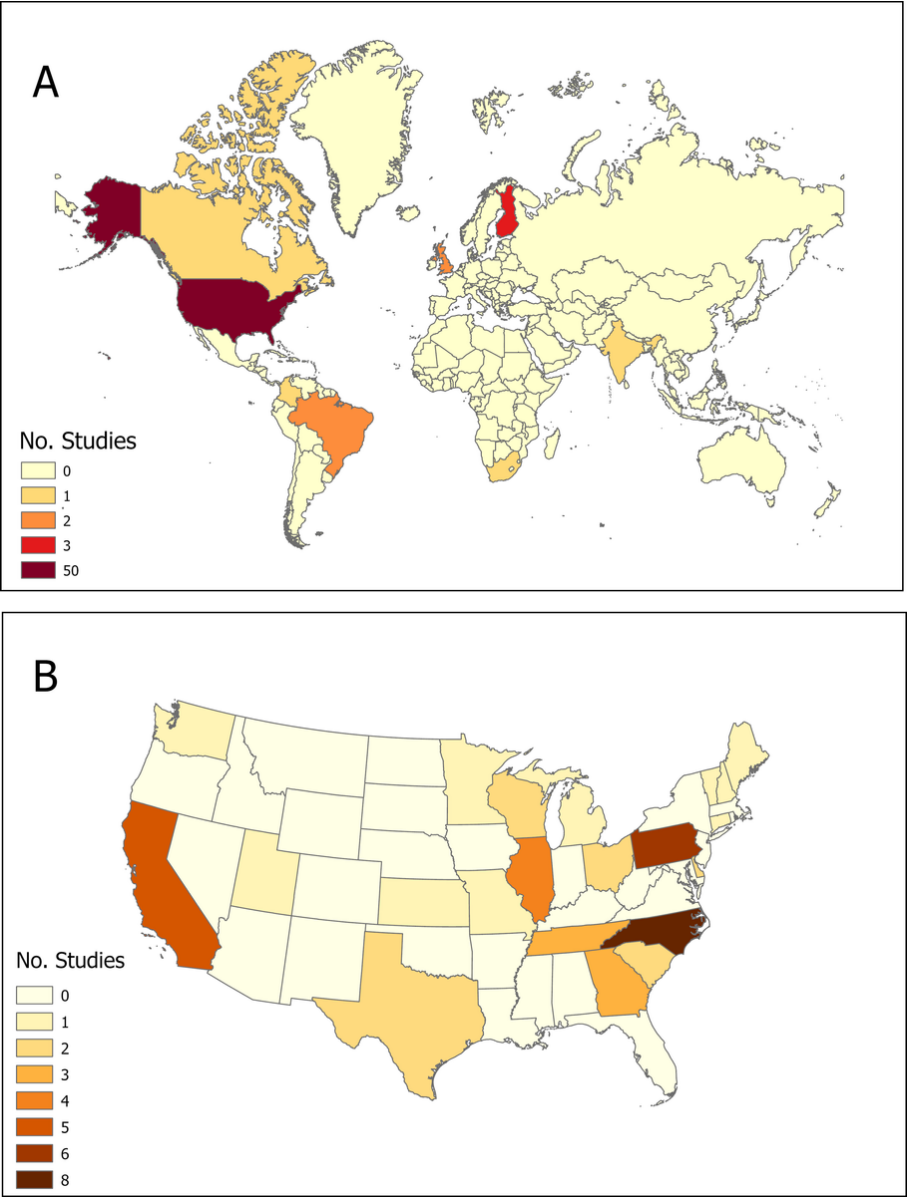
Geospatial distribution of the included studies (A) Worldwide and (B) at the state level in the US

While no time restrictions were imposed, a limited number of articles (n = 11, 17.7%) were published prior to 2017. The earliest article included in this study was published in 2009, and the publication frequency has experienced a significant upsurge since 2017 (n = 51, 82.3%). Moreover, despite no geographic limitations, distinct geographic disparities were evident. The articles were reported from only nine countries, with the majority from the US (n = 50, 80.6%), followed by Finland (n = 3, 4.8%), the United Kingdom (n = 2, 3.23%), Brazil (n = 2, 3.23%), with Canada, Columbia, India, the Netherlands, and South Africa each contributing one article. There were only two articles at the national level. General characteristics of the included articles are presented in Supplementary Appendix S2. Most US-based studies were concentrated in North Carolina (n = 8, 16%), Pennsylvania (n = 6, 12%), California (n = 5, 10%), and Illinois (n = 4, 8%). Figure 2 illustrates the geospatial distribution of articles worldwide (A) and at the state level in the US (B).

### Spatial methodologies

Most studies focused on frequentist methods compared to the Bayesian methods. Among those, the most prevalent category was clustering (n = 34), followed by descriptive (n = 17), modeling (n = 8), and spatiotemporal analyses (n = 2).

#### Descriptive analyses

Descriptive analyses were categorized into four groups: spatial sampling (n = 2), spatial overlay (n = 2), proximity analysis (n = 8), and spatial interpolation (n = 5).

### Spatial sampling

A two-standard deviation ellipse method is employed to optimize spatial sampling density that contains almost 95% of the locations of patients [11]. [12, 13] adopted this approach when sampling women who underwent cytomegalovirus antibody testing during pregnancy, especially in peripheral areas with limited subject representation.

### Spatial overlay

Spatial overlay integrates various spatial data sources, often maps, to represent their shared features. [14] overlaid the map of major radiation treatment interruptions based on race onto the map of median household income. [15] spatially joined patient addresses to the nearest city parcels and computed an estimate of the incidence of emergency department visits for asthma for each parcel.

### Proximity analysis

Proximity analysis includes measuring distances between geographic features to identify nearby features within a defined distance or buffer zone to uncover proximity patterns [16]. [17] created temporal and spatial buffers to assess the correlation between individual exposure to violent crime and blood pressure. [18] utilized spatial buffers and found that most patients with adenomas larger than 25 mm lived more than 20 km from medical centers. [19] evaluated the associations between environmental factors and body mass index (BMI) within a 0.5-mile network buffer from the place of residence. [20] investigated the associations between prenatal residential greenness and birth outcomes within 250m and 1,250m buffers. [21, 22] used GIS network analysis, specifically an origin-destination cost matrix, and estimated travel time and expenses between the atrial fibrillation patient’s residential address and the location of healthcare facilities providing anticoagulant therapies. Utilizing a GIS service area network analysis, [23] examined BMI percentile and proximity to fast-food and pizza establishments among adolescents within 0.25 mile Euclidean and network buffer zones. [24] created network flow maps calculated the distances between clinic transfers and found concentrated participant transfers in certain urban areas.

### Spatial interpolation

Ordinary Kriging is one of the most widely used spatial interpolation techniques that leverages the spatial autocorrelation structure of observed locations to estimate values at unmeasured locations [25]. [26] applied ordinary Kriging with a spherical semi-variogram model based on observations of the child elevated blood lead level (BLL) geocoded to the home address to visualize BLL variations before and after water source changes. [27] interpolated the levels of neighborhood physical disorder based on an exponential variogram. [28] demonstrated spatial variations for the incidence rates of each ICD-9 diagnostic code based on an exponential variogram. [29] estimated monthly average concentrations of fine particulate matter to investigate the associations between air pollution exposure during pregnancy and gestational diabetes mellitus (GDM). [30] generated heat map for type 2 diabetes per general practice via ordinary Kriging.

#### Spatial clustering

Spatial clustering techniques assess whether health outcomes are random, uniform, or clustered and pinpoint the locations of clusters [31]. Spatial clustering was the most widely used category (n = 34) among all studied categories. Moran’s I clustering and cluster detection were the most frequent techniques (n = 17), followed by kernel/point density estimation (n = 7), spatial scan statistics (n = 6), and Getis-Ord Gi* statistics (n = 4).

### Kernel/point density estimation

Kernel density estimation (KDE) generates a smooth surface to visualize areas of the most significant spatial intensity by calculating a distance-weighted count of events within a specified radius per unit area [32]. Several studies adopted KDE to analyze patterns, including cholera hospitalization [33], extended-spectrum β-lactamase phenotypes [34], human Sporotrichosis [35], comparison of the spatial intensity of chronic kidney disease (CKD) with non-CKD patients [36], and comparison of the spatial intensity of breast cancer and non-breast cancer [37]. Using the point density function, [38] pinpointed hotspots of inpatient bed-day rates within a 2-mile radius of a medical center and [39] estimated the number of participants per square mile.

### Global and local Moran’s I

Global Moran’s *I* (GMI) evaluates the overall pattern for spatial autocorrelation [40] by inferring if a variable is spatially clustered or over-dispersed vs. being randomly distributed under the null hypothesis [40]. Local Moran’s *I,* often called LISA, is used to locate statistically significant clusters, including hotspots, cold spots, and outliers [41]. GMI has been adopted to analyze spatial clustering of health outcomes, including GDM [29], day-of-surgery cancellation [42], obesity [43], and COVID-19 [44]. All exhibited clustered patterns. [33] analyzed three groups: depression, obesity, and comorbid cases, confirmed clustering for all outcomes, and identified spatial clusters and outliers. [45] found random distributions for dermatomyositis (DM) and subtypes, classic DM (CDM), and clinically amyopathic DM (CADM). Using LISA [46] located eleven clusters with high amyotrophic lateral sclerosis incidence rates. Meanwhile, [47] pinpointed clusters with higher or lower depression prevalence, and [48] identified a cluster of low utilization of acute pediatric mental health interventions in less-densely populated rural border areas.

GMI and (semi)variograms can also identify spatial autocorrelation in model residuals. If detected, the models are adjusted accordingly to avoid biased estimates. For example, [49] modeled nontuberculous mycobacteria (NTM) disease, shifting from a non-spatial Bayesian model to a spatial model when spatial autocorrelation was found in residuals. Similarly, [50] incorporated spatial random effects into a prostate cancer model due to significant GMI in the residuals. [51] used variograms to assess spatial dependency in cleft lip and/or palate, leading to a geostatistical model over standard logistic regression. Conversely, [20] found no spatial autocorrelation in non-spatial model residuals.

The bivariate GMI quantifies the overall spatial dependence between two distinct variables (positive value indicates high values of one variable are surrounded by high values of the other or low values are surrounded by low values, while negative value implies high values of one variable are surrounded by low values of the other) [52]. Bivariate LISA assesses the relationship at the local level. [45] employed bivariate GMI for the prevalence of DM, CDM, and CADM with airborne toxics but found no overall spatial dependencies. However, bivariate LISA identified local dependencies at the zip code level. [53] applied bivariate GMI and found significant overall associations between longer (average) distances to the nearest supermarket and higher incidence of diabetes, and bivariate LISA identified significant “high-high” relationships at the zip code level. [54] utilized bivariate LISA and found no local association between radiation therapy interruption and social vulnerability index at the zip code level.

### Getis-Ord Gi*

The Getis-Ord Gi* statistic identifies high or low-value clusters (hotspots and cold spots) by assessing deviations of health outcomes at locations from the average within a defined neighborhood [55]. [56] measured racial residential segregation by examining the deviations in the (proportion of) African American residents in each census tract from the mean of neighboring tracts. Similarly, [57] measured the racial residential segregation for the percentage of non-Hispanic Black residents. [7] identified significant community-onset methicillin-resistant Staphylococcus aureus (CO-MRSA) hotspots with distinct patterns between cases and controls. [58] detected the high and low values clusters for the child opportunity index and median household income.

### Spatial scan statistics

Spatial scan statistics technique identifies high and low-risk clusters and estimates their relative risks [59]. It also can incorporate covariates to characterize underlying patterns [60]. [49] found that people living in zip codes within the primary cluster had an almost 2.5 times greater risk of NTM disease. [61] identified clusters of under-immunization and vaccine refusal among children, with rates ranging from 18–23% inside the clusters compared to 11% outside. [62] identified high and low-risk clusters of heart disease primarily in rural areas, with smaller and fewer clusters after adjusting for age. [63] found that different case definitions had no significant impact on the locations of geographic asthma clusters.

The technique can also pinpoint cold spots. [64] identified areas with significantly lower COVID-19 testing than expected, indicating a need for interventions. [65] observed significantly low rates of up-to-date colorectal cancer screening.

#### Spatial modeling (frequentist)

Among the included articles, the generalized additive models (GAMs) emerged as the most frequently employed spatial models. GAMs can account for spatial autocorrelation by incorporating smooth functions (such as thin-plate regression) of spatial coordinates [66], allowing the estimate of geographic variation with or without covariate adjustments. GAMs identified spatial variabilities in asthma prevalence [3, 67] and cytomegalovirus [12, 13], although such variations often diminished when adjusted for demographic factors such as race and age. Among less commonly used geospatial models were spatial trend [68], generalized linear mixed effects [44], and spatial error [42] models.

#### Spatiotemporal analysis

Only two studies explored spatiotemporal patterns, and no spatiotemporal modeling was conducted. [69] employed space-time scan statistics to study the spatiotemporal patterns of childhood asthma and found a significant frequency increase (2009–2013) and a rising trend from 4 to 16 per 1,000 children (2005–2015). [7] employed the space-time cube tool and emerging hotspot analysis to analyze the spatial-temporal trends and evolving patterns of CO-MRSA from 2002 to 2010. They identified several types of space-time hotspots of CO-MRSA, including new, consecutive, intensifying, sporadic, and oscillating hotspots.

#### Bayesian analysis

The articles employing Bayesian methods were categorized into Empirical Bayes smoothing (n = 5) and Bayesian modeling (n = 6).

The Empirical Bayes smoothing was employed in [33, 42, 43, 56] to stabilize estimated rates in areas with limited data points by borrowing information from the overall population [70]. [71] employed non-parametric kernel smoothing to estimate the prevalence of childhood obesity in areas with sparse observations (n < 20 individuals).

Bayesian modeling can account for spatial and temporal dependencies and quantify uncertainty by specifying prior distributions [72]. Among the articles, the conditional autoregressive (CAR) prior emerged as the most used, with two variants: intrinsic and multivariate CAR. Intrinsic CAR assessed the spatial variations in: diabetes in relationship with racial isolation [73], hypertension related to racial isolation [74], and type 2 diabetes mellitus with the built environment [75]. Multivariate CAR was employed to identify areas with higher or lower-than-expected prostate cancer while controlling for risk factors [50]. Moreover, hierarchical Bayesian that can incorporate hierarchical structures for modeling interactions in data with multiple levels [76] was used to investigate spatial distributions of patients admitted for drug-related reasons concerning the area deprivation index [77]. Bayesian negative binomial hurdle models that can account for excessive zeros and overdispersion were used by [78] to examine spatial variation between patient responses to the questions concerning unhealthy home environments and the mean number of emergency department visits after screening.

### Phenotyping

**Figure 3:**
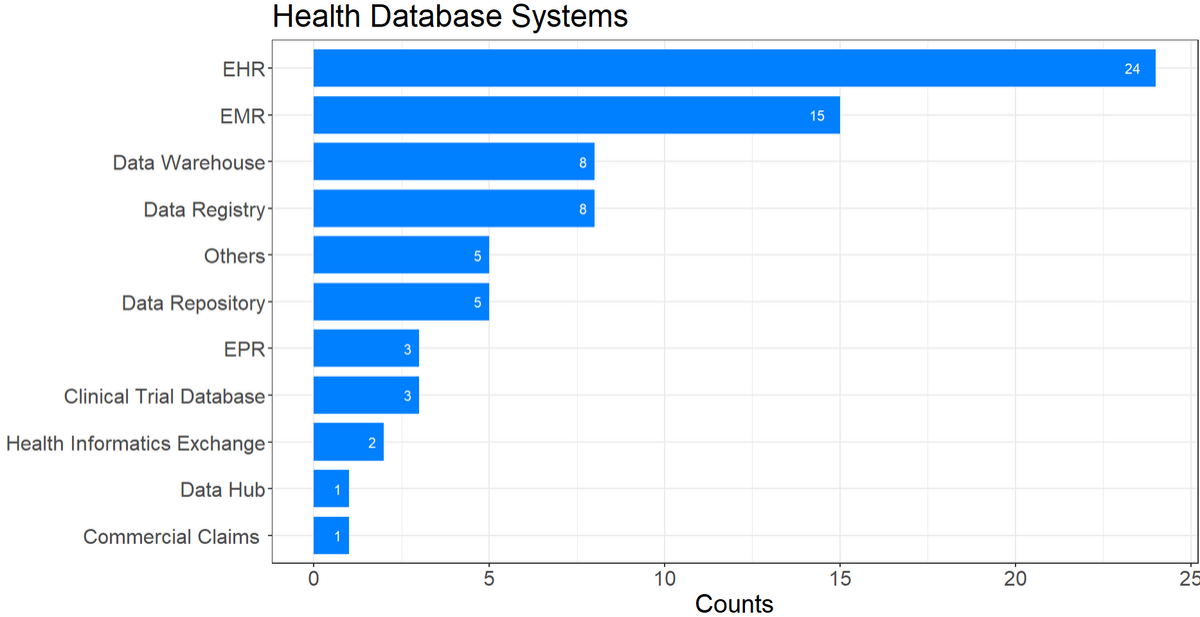
Health database systems used in each publication. The numbers indicate how often each database system was utilized, with the caveat that a study may have used more than one system.

Most publications relied on a single health information system (n = 53), and some utilized two (n = 10). It is important to note that a study may have used multiple systems. According to Fig. 3, EHR systems (n = 24) were the most used, followed by EMR systems (n = 15), data warehouses (n = 8), registries (n = 8), and repositories (n = 5). Other systems, like those for clinical trials (n = 3), health information exchanges (HIE) (n = 2), national commercial claims datasets (n = 1), and data hubs (n = 1), were less frequently used. In five articles, the database systems weren’t explicitly mentioned in the publication, supplementary materials, or references. We have categorized these as “Others” in Fig. 3.

#### Clinical domain characteristics and themes

The largest category of articles was classified under the infectious disease (n = 13), followed closely by endocrinology (n = 10) and cardiology (n = 10) domains. Additionally, 20 articles had a pediatric domain or focus, as noted with an additional column in Supplementary Appendix S3. Maternal and newborn care was classified as its own domain (n = 7), but it overlapped with other domains such as pediatrics, cardiology, endocrinology, and infectious disease.

The relationship between the clinical domains and the “conditions/problems of focus” in each article was examined (Supplementary Appendix S3). In some cases, direct correspondence was observed, while in other instances, the “condition/problems of focus” differed from the phenotype of the patient cohort. In many articles, one or more overlapping domains were observed (e.g., rheumatology, neurology, and dermatology for the study of dermatomyositis). Asthma, hypertension, and diabetes were studied most frequently (n = 6 for each). Four articles did not focus on any health condition but rather on examining disparities in either a data source or a specific domain or cohort (e.g., disparities in the use of pediatric intensive care units).

Every article was attributed to at least one prominent theme, with the possibility of multiple themes. SDOH themes were prevalent in many articles. To organize and present this information, we utilized the domains defined by the Healthy People 2030 framework. There are five domains in the SDOH framework (Table 1), with the corresponding counts of these domains being seen as themes of the articles. Most articles had one or more SDOH themes (n = 55). Many articles focused either on all the domains or SDOH holistically without particular focus on any specific domain (n = 36). However, some articles contained prominent themes that were not directly related to SDOH, which were phenotypic features (n = 7), followed by ecological (n = 5), environmental (n = 5) with climate, genomics, and microbiome, each contributing one article.

#### Clinical phenotype features

For each publication, clinical phenotype definitions were extracted (Supplementary Appendix S4). In almost all studies, phenotype definitions included demographic details such as patient age, race, and gender, along with some diagnostic characteristics (e.g., asthma diagnosis). Only a limited number of phenotypes were observed to be validated (n = 9 articles). The most frequently observed method for phenotype validation was a manual chart review of all matches or a sample of matched charts. None of the articles with chart review as a validation method shared information on the match rate. Additionally, only two articles [33, 75] were observed to utilize validated eMERGE Network computable phenotypes from the Phenotype Knowledgebase (PheKB) [79–81].

## DISCUSSION

This systematic review is the first comprehensive investigation of spatial methodologies within HIS-derived data. Spatial clustering and descriptive analysis were the most used methods, while space-time modeling, either frequentist or Bayesian, remained under-explored. The diverse use of spatial analysis for HIS-derived data in different health domains highlights the potential to incorporate spatial methods to enhance the context of individual patients for future biomedical research. We found a limited global use of HIS-derived data for spatial analysis, involving only nine countries, possibly due to incomplete HIS adoption or data availability. Surprisingly, no studies were conducted in countries with well-established HIS [82], like most European countries. The scarcity of published articles is primarily due to the challenge of safeguarding patient privacy. Address data, crucial for spatial analysis, is highly confidential and often restricted from sharing. Researchers and institutions often use geographic masking techniques [6, 83] to balance data utility and privacy protection by altering the precise geographic coordinates while preserving the overall spatial characteristics of data. Encouraging spatial analysis adoption in these countries could promote biomedical knowledge sharing and collaboration.

The application of spatiotemporal analysis of HIS-derived data was mainly limited to exploring spatiotemporal clusters with no spatiotemporal modeling. This might be due to the technical expertise required for analysis, data complexity, availability of longitudinal data, and computational challenges. However, spatiotemporal modeling can aid in understanding disease trends and progressions, seasonality, and long-term shifts at the local levels [84]. Moreover, they offer a more flexible framework to handle complex spatial and temporal dependencies, control confounding variables [85], and incorporate prior information, such as existing medical literature and expert opinions, resulting in more interpretable results [86, 87]. Bayesian modeling can better account for uncertainty in parameter estimates and predictions to assess the reliability of findings before implementing interventions [88]. Future research in this field should delve into spatial and spatiotemporal modeling, focusing on Bayesian approaches.

Among the health conditions studied, chronic and infectious diseases emerged as the most frequently investigated domains compared to others. This disparity may be attributed to the pressing public health concerns posed by diseases with immediate impacts that often attract more funding and resources for research initiatives [82]. The historically high mortality rates of these conditions likely led to continuous research. Surprisingly, despite the plethora of funding in cancer research, we only found six articles within the cancer domain, which may likewise be attributed to and indicative of the pressing needs of other domains, such as infectious disease.

We observed recurring and prominent themes related to the SDOH. This emphasis may result from the growing maturity and increased awareness within the biomedical informatics community regarding the significant influence of social, economic, and environmental factors on health outcomes. Understanding the roles of SDOH in health disparities will likely lead to the implementation of integrative health interventions that address the needs of individuals affected by these health disparities. These interventions can likewise incorporate spatial perspectives.

Our review found only two HIE systems, which was unexpected given the inherent capabilities of these systems to effectively harness the power of spatial data. The robustness of HIEs, along with their diverse patient base from multiple institutions, enables them to offer insights that are not only comprehensive but also more nuanced. The significant scarcity of HIEs in the literature underscores a potential opportunity for leveraging these platforms in cutting-edge health informatics research. By integrating spatial data and analysis, these platforms can become valuable resources for researchers exploring and addressing SDOH and health disparities.

Another missed opportunity is the underutilization of computable phenotypes – automated algorithms designed for characterizing diseases and enrolling patients in studies. Most studies primarily depended on the manual application of inclusion and exclusion criteria to define phenotypes. While this method may be suitable in certain scenarios, it often necessitates greater depth and granularity to consistently and accurately capture the intended patient cohorts. The accuracy and precision of the manual approach can vary depending on the data sources and clinical domains. Notably, only two of the studies in our review used computable phenotypes, indicating a significant underutilization of this essential and potentially transformative approach, highlighting a noteworthy area for growth. Furthermore, only five articles carried out any form of chart review validation. Validation methods, including chart reviews, genetic markers, and clinical variables, are indispensable in phenotyping to guarantee the accurate characterization of the desired cohorts. This applies even to computable phenotypes within specific medical domains [89].

This study has several main limitations. First, we only considered English articles, possibly introducing language bias. Additionally, selection bias is possible due to database availability. However, we mitigated these limitations by searching Google Scholar and conducting backward reference checking to identify relevant studies that might yet be identified through our initial search strategy. Lastly, we used a query search strategy with the keywords limited to HIS, including EHR, EMR, EPR, EDW, and RDW. However, this approach inherently restricts the scope of articles we retrieve, potentially omitting studies that do not utilize these specific terms in their abstract or title. For a more encompassing future exploration, it would be judicious to expand the list of keywords to include broader terms such as claims data, research consortium data (as defined appropriately), data hubs, enclaves, registries, and repositories.

## CONCLUSION

This systematic review provided a comprehensive overview of the current utilization of spatial analysis in HIS-based research and underscored the pivotal role that spatial analysis can play in addressing complex health problems. The utilization of HIS-derived spatial analysis is on an upward trajectory, parallel with the widespread adoption of HIS systems. The volume of articles on this topic is anticipated to continue to grow. We found limited global use of HIS data for spatial analysis, with participation limited to nine countries. This review also highlighted the need for additional exploration of spatial analysis techniques, including but not limited to spatiotemporal Bayesian analysis and modeling, particularly in the cancer domain.

## Figures and Tables

**Table 1:** SDOH themes examined within the framework of Healthy People 2030 SDOH domains.

Labels	SDOH Domains	Counts
SDOH 1	Economic Stability (Employment, Food Insecurity, Housing Instability, Poverty)	2
SDOH 2	Education Access and Quality (Early Childhood Dev and Ed, Enrollment in Higher Ed, HS Graduation, Language and Literacy)	NA
SDOH 3	Health Access & Quality (Access to Health Services, Access to Primary Care, Health Literacy)	5
SDOH 4	Neighborhood and Built Environment (Access to Foods that Support Healthy Dietary Patterns, Crime and Violence, Environmental Conditions, Quality of Housing)	14
SDOH 5	Social and Community Context (Civic Participation, Discrimination, Incarceration, Social Cohesion)	5
All 5 SDOH domains *or* SDOH as a whole		36
Non-SDOH focus		8
